# Cytomegalovirus-specific CD8+ T-cells are associated with a reduced incidence of early relapse after allogeneic stem cell transplantation

**DOI:** 10.1371/journal.pone.0213739

**Published:** 2019-03-19

**Authors:** Pavankumar Reddy Varanasi, Justyna Ogonek, Susanne Luther, Elke Dammann, Michael Stadler, Arnold Ganser, Sylvia Borchers, Lothar Hambach, Eva M. Weissinger

**Affiliations:** 1 Department of Hematology, Hemostasis, Oncology and Stem Cell Transplantation, Hannover Medical School, Hannover, Germany; 2 German Center for Infection Research (DZIF) Hannover Medical School, Hannover, Germany; 3 RHEACELL GmbH & Co. KG, Heidelberg, Germany; Hospital Infantil Universitario Nino Jesus, SPAIN

## Abstract

Leukemia relapse is the main cause for mortality after allogeneic stem cell transplantation (allo-SCT). Donor-derived allo-immune responses eliminate the residual host hematopoiesis and protect against relapse. Cytomegalovirus (CMV) reactivation (CMV-R) after allo-SCT may trigger anti-leukemic effects. The impact of CMV-specific CD8+ T-cells (CMV-CTLs) on the outcome after allo-SCT is currently unknown. Here, we studied the relationship between CMV-CTLs, overall T-cell reconstitution and relapse incidence in 103 patients with acute leukemia (n = 91) or myelodysplastic syndrome (n = 12) following CMV-seropositive recipient/donor (R+/D+) allo-SCT. Patients were subdivided based on the presence or absence of CMV-CTLs at 3 months after allo-SCT. Presence of CMV-CTLs was associated with preceding CMV-R and a fast T-cell reconstitution. Univariate analysis showed a significantly lower 1-, 2- and 5-year cumulative incidence of relapse (CIR) in patients with CMV-CTLs compared to those without CMV-CTLs. Multivariable regression analysis of the outcome performed with other relevant parameters chosen from univariate analysis revealed that presence of CMV-CTLs and chronic graft-versus-host disease (cGvHD) were the only independent factors associated with a low CIR. Onset of relapse was significantly later in patients with CMV-CTLs (median 489 days) than in in those without (median 152 days, p = 0.041) during a five-year follow-up. Presence of CMV-CTLs was associated with a lower incidence of early relapses (1 and 2-years), while cGvHD lead to a lower incidence of late relapses (2 to 5-years). In conclusion, our data show that CMV-CTLs indicate a functional immune-reconstitution protective against early relapse.

## Introduction

Relapse is the main cause for mortality after allogeneic stem cell transplantation (allo-SCT) in patients with acute leukemia and myelodysplastic syndrome (MDS) [1]. An adverse disease status [2, 3], unfavorable cyto- and molecular-genetics [4, 5] or reduced intensity conditioning (RIC) [6] are major disease or transplant related risk factors for relapse after allo-SCT. The immune-mediated graft-versus-leukemia (GvL) effect after allo-SCT is often associated with the occurrence of graft-versus-host disease (GvHD) [1]. Chronic but not acute GvHD has been shown to be protective against relapse of acute leukemia [7] and myelodysplastic syndrome (MDS) [8]. The exact mechanisms driving the allo-immune responses responsible for the GvL effect and for GvHD are still unknown.

Recent studies provide increasing evidence that cytomegalovirus (CMV) influences allo-immune responses after allo-SCT. CMV reactivation (CMV-R) has been described to boost the overall T-cell reconstitution [9, 10] and to be associated with GvHD [11, 12]. However, the impact of CMV-R on the protection against relapse is still highly controversial. While some studies demonstrated a reduced leukemia relapse risk in patients after CMV-R [13–15], others reported no impact of CMV-R on the relapse incidence after allo-SCT [16–18]. Nevertheless, two recent studies showed that host chimerism is considerably influenced by the CMV specific donor immunity. CMV-seropositive patients showed lower host chimerism levels subsequent to reduced intensity conditioning (RIC) when transplanted from a CMV-seropositive (R+/D+) as compared to CMV seronegative donors (R+/D-) [19]. Moreover, we have shown recently that patients transplanted in the CMV R+/D+ setting after RIC have a faster overall T-cell reconstitution and lower host chimerism levels in the presence of CMV-CTLs at 3 months after allo-SCT [20]. Persistence of complete donor chimerism is an important indicator for complete remission (CR) after allo-SCT [21, 22]. Since alloreavtiveT-cells are responsible for the conversion to complete donor chimerism [23], our data provided first evidence that CMV-CTLs may not only trigger the reconstitution of T-cells but also allo-immune responses in the CMV R+/D+ setting. To date, it is unclear whether this proposed effect of CMV-CTLs on allo-reactivity also translates in a reduced relapse incidence in the long term follow-up of patients.

Here, we studied the relationship between CMV-CTLs, overall T-cell reconstitution and relapse incidence in patients with acute leukemia or MDS after allo-SCT in the CMV R+/D+ setting.

## Patients, materials and methods

### Patient cohort

All CMV-seropositive patients transplanted with a T-cell replete graft of a CMV-seropositive donors between May 2006 and December 2014 at the Hannover Medical School were eligible for this study. Myeloablative conditioning (MAC) regimens were based on busulfan (Bu, n = 17) and total body irradiation (TBI, n = 16). Reduced intensity conditioning (RIC) was preceded by additional anti-leukemic treatment in 40 patients using FLAMSA [24] (n = 28) or ClArac [25] (n = 12). RIC protocols comprised busulfan (n = 27), melphalan (n = 23) and TBI (n = 20), based protocols. T-cell depletion was achieved either with antithymocyte globulin (ATG-F (n = 68, Fresenius Biotech, Gräfelfing, Germany) or Thymoglobuline (n = 24, Genzyme, Naarden, The Netherlands). Patients were typed for 10 HLA alleles on high resolution level for exon 2+3 for HLA-A, B, C and for exon 2 for HLA-DRB1 and -DQB1 according to the current European Federation for Immunogenetics guidelines. Donors typed on high resolution level were considered HLA-matched, if they were identical for 10/10 HLA-alleles to the respective patients. Sibling donors were considered HLA-matched also when identical for HLA-DRB1 and -DQB1 typed on high resolution level and phenotypically identical for HLA-A and–B. Patients receiving haploidentical or cord blood ALLO-SCT were excluded from the study. ALLO-SCT protocols were approved by the Institutional Review Board of Hannover Medical School. Informed consent was obtained in writing from all patients in accordance with the Helsinki declaration. The analysis was approved by the Institutional Review Board of the Hannover Medical School (1886–2013 and 2934–2015).

### T-cell monitoring

T-cell immune reconstitution was monitored in peripheral blood samples at 1, 2 and 3 months after allo-SCT as previously described [20]. Briefly, fresh whole blood was stained with anti-CD3, anti-CD8 and either anti-CD4 antibodies (all from Beckman Coulter, Marseille, France) or one of 6 commercially available HLA/CMV tetramers (HLA-A*01:01 pp50-VTEHDTLLY; HLA-A*02:01 pp65-NLVPMVATV; HLAA*24:02 pp65-QYDPVAALF; HLA-B*07:02 pp65-TPRVTGGGAM; HLA-B*08:01 IE1-ELRRKMMYM; HLA-B*35:01 pp65-IPSINVHHY, MBL International, Woburn, USA). The tetramers containing the A245V mutation in the HLA class I heavy chain α3 domain were selected due to their reduced background staining [26]. The HLA-A*02:01/negative tetramer loaded with a proprietary non-antigen related peptide (PE, MBL) was used as negative control. After tetramer staining at room temperature (RT) for 30 mins, erythrocyte lysis was performed as previously described[27]. After standardization using calibration beads, samples were acquired on a FC500 flow cytometer (Beckman Coulter). Fluorescent beads (FlowCount Fluorospheres, Beckman Coulter) were used to determine absolute T-cell numbers. CMV-CTL numbers for every tetramer were calculated: CMV-tetramer binding T-cells minus negative-control-tetramer binding T-cells. The CMV-CTL levels were calculated as mean of CMV-CTL counts obtained for each tetramer used. CXP software (Beckman Coulter) was used for FACS.

### Clinical parameters and events

**Advanced (in contrast to standard) disease status** was defined as acute myeloid leukemia (AML) beyond first cytological remission or persistent disease after second induction therapy, acute lymphoblastic leukemia (ALL) beyond first cytological / molecular remission or persistent disease after second induction therapy, high-risk myelodysplastic syndrome (MDS) (IPSS higher than intermediate-2) and CML blast crisis. **Adverse (in contrast to standard) cyto- and molecular genetics** was defined for AML according to the ELN adverse risk [28], for ALL by the presence of t(9;22) or t(4;11) or a complex karyotype (≥ 3 anomalies), for MDS by the presence of a complex karyotype or chromosome 7 anomalies and for bcr-abl positive CML by the presence of additional molecular abnormalities. **Relapse** was defined as detection of leukemic blasts in the peripheral blood or of more than 5% blasts in the bone marrow, as detection of multi-lineage dysplasia in the bone marrow (in cases of MDS or unexplained by concurrent medication) or as extramedullary disease manifestation. None of the patients had relapse prior to the month 3 measurement of CMV-CTLs. **CMV reactivation** was detected by monitoring of peripheral blood samples for CMV-DNA during aplasia followed by measurement of CMV-pp65 antigen in leukocytes [27]. CMV reactivation was defined as 1) CMV-DNA load increase by more than 0.5 log levels above the baseline, 2) more than 5 pp65 antigen positive cells per 4x10^5^ leukocytes in a single test or more than 2 pp65 antigen positive cells per 4x10^5^ leukocytes in 2 consecutive tests. CMV reactivation was preemptively treated first line with ganciclovir and second line with foscarnet. **Acute GvHD** (aGvHD) was graded according to the Glucksberg score [29]. **Chronic GvHD** (cGvHD) was diagnosed and staged according to the Seattle criteria [30].

### Statistical analysis

Major study endpoints were overall survival (OS), disease-free survival (DFS), NRM (non-relapse mortality) or cumulative incidence of relapse (CIR). Kaplan-Meier curves were used to estimate the probability of OS, DFS and the curves were compared by the log-rank test [31]. Time to death after allo-SCT was considered as an event for OS and time to death or time to relapse was considered as an event for DFS. The CIR and NRM were compared by Gray´s test in a competing risk setting [32]. For relapse, NRM was considered as a competing risk factor and vice versa. The categories reaching a p-value below 0.05 were included in the multivariable Cox proportional hazards regression model. Potential factors affecting OS and DFS outcomes were identified by multivariable analyses using Cox proportional hazards regression models [33]. The NRM and CIR were estimated by the proportional subdistribution hazard regression model of Fine and Gray [34]. Continuous variables were analyzed by Mann-Whitney U test. Statistical analysis was performed using the Statistical Program for Social Science (SPSS version 23, IBM, New York, USA), and EZR [35] on Rcommander (R-software ver. 3.4.1, http://www.R-project.org). A p-value below 0.05 was considered statistically significant. Figures on T-cell reconstitution were prepared with GraphPad Prism 6 (California, USA).

## Results

### Patient cohort

Patients (n = 103) without relapse until month 3, with data on CMV-CTL recovery at month 3 and a minimum follow-up of 2 years after allo-SCT were included in this analysis. Patients were subdivided in two groups based on the presence or absence of more than 1 CMV-CTLs per μL blood 3 months after allo-SCT. This threshold was based on our previous observation that more than one CMV-CTL/μl blood at 3 months after allo-SCT was associated with low host chimerism levels[20]. CMV-CTLs were detected in 87/103 patients (84%) 3 months after allo-SCT. Detailed patient characteristics are shown in **Table 1**. The demographic and clinical parameters were not significantly different in patients with or without CMV-CTLs **(Table 1)**.

**Table 1:** Patient characteristics.

	CMV-CTL neg. n = 16	CMVCTL pos. n = 87	p value
	No. (%)	No. (%)	
**Median patient age (range)**	59 (22–72)	52 (19–70)	0.202
**Median donor age (range)**	44 (16–58)	39 (20–68)	0.588
**Recipient gender**			0.585
Male	11 (69)	52 (60)	
Female	5 (31)	35 (40)	
**Diagnosis**			0.330
AML	10 (62.5)	64 (73.6)	
ALL	2 (12.5)	13 (15)	
MDS	3 (19)	9 (10.3)	
Others^a^	1 (6)	1 (1.1)	
**Disease status**			0.588
standard	8 (50)	51 (59)	
advanced	8 (50)	36 (41)	
**Cyto- and molecular genetics**			0.782
standard	9 (56)	54 (62)	
high risk	7 (44)	33 (38)	
**Stem cell source**			0.497
PBSC	15 (94)	84 (97)	
BM	1 (6)	3 (3)	
**Donor**			0.695
MRD	3 (19)	25 (29)	
MUD	11 (69)	48 (55)	
MMUD	2 (12)	14 (16)	
**Conditioning**			1.00
MAC	6 (37.5)	34 (39)	
RIC	10 (62.5)	53 (61)	
**GvHD prophylaxis**			0.755
CSA/MMF	13 (81)	64 (74)	
CSA/MTX	3 (19)	23 (26)	

Statistical analysis was performed to compare patient characteristics between CMV-CTL negative (neg.) and CMV-CTL positive (pos.) patients. Comparisons of patient and donor age were performed by Mann-Whitney U test. Comparisons of recipient gender, disease status, cyto- and molecular genetics, stem cell source, GvHD prophylaxis and conditioning regimen were performed using Fisher’s exact test. Comparisons of diagnosis, donor and T-cell depleting antibodies were performed using chi-square test. Immunosuppressive antibodies (antithymocyte globulin (ATG, Fresenius) or Thymoglobulin, Genzyme) for in vivo depletion of T-cells were given to 91 patients (88%), while only 12 were not treated with in vivo T-cell depletion. Fifteen patients (93%) without CMV-CTL and 77 (88.5%) with CMV-CTL received ATG or Thymoglobulin. **Abbreviations:** No., number; %, percentage; AML, acute myeloid leukemia; ALL, acute lymphoblastic leukemia; MDS, myelodysplastic syndrome; PBSC, peripheral blood stem cells; BM, bone marrow; MRD, Matched related donor; MUD, Matched unrelated donor; MMUD, Mismatched unrelated donor; MAC, myeloablative conditioning; RIC, reduced intensity conditioning; CSA, Cyclosporine A; MMF, mycophenolate mofetil; MTX, methotrexate

^a^: AL; biphenotypic acute leukemia (n = 1); CML, chronic myeloid leukemia blast crisis (n = 1).

### Immune reconstitution, CMV reactivation, acute and chronic GvHD

**Fig 1:**
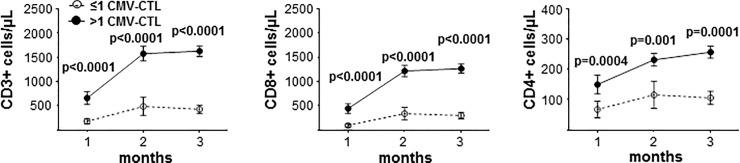
Immune reconstitution is dependent on the presence of CMV-CTLs. Course of CD3+ (left), CD8+ (middle) and CD4+ (right) T-cell reconstitution at 1, 2 or 3 months dependent on the presence (filled circles) or absence (open circles) of CMV-CTLs at the respective time points after allo-SCT in the CMV R+/D+ setting. The data points indicate mean ± standard error of the mean (SEM). Statistical analysis was performed using Mann-Whitney U test, P-values <0.05 are indicated.

Patients were monitored for the reconstitution of overall T-cells and CMV-CTLs at 1, 2 and 3 months after allo-SCT. Patients with CMV-CTLs had significantly more CD3+, CD8+ and CD4+ T-cells at these time points than patients without CMV-CTLs (Fig 1). Next, the incidences of CMV reactivation (CMV-R), clinically significant aGVHD grade II-IV and cGvHD were analyzed depending on the presence or absence of CMV-CTLs at 3 months after allo-SCT. CMV-R occurred in 63 patients (61%) on day 36 (median; range 8 to 68) after allo-SCT. The incidence of CMV-R was higher in patients with CMV-CTLs than in those without (67% vs. 31%; p = 0.011). Acute GvHD grade II-IV occurred in 27 patients (26%) on day 39 (median; range 18–123) after allo-SCT irrespective of the detection of CMV-CTLs (26% vs 25%, p = 1.0). Chronic GvHD developed in 41 patients (40%) on day 167 (median; range 94–809) after allo-SCT independent of the presence of CMV-CTLs (42.5% vs 25%, p = 0.268) **(Table 2)**.

**Table 2:** Complications after allo-SCT.

Complications	CMV-CTL neg. n = 16	CMV-CTL pos. n = 87	p value
	No. (%)	No. (%)	
**CMV-R**			**0.011**
no	11 (69)	29 (33)	
yes	5 (31)	58 (67)	
**aGvHD**			1.00
grade 0-I	12 (75)	64 (74)	
grade II-IV	4 (25)	23 (26)	
**cGvHD**			0.268
no	12 (75)	50 (58)	
yes	4 (25)	37 (42)	

Statistical analysis was performed to compare complications between CMV-CTL negative (neg.) and CMV-CTL positive (pos.) patients. Comparisons of CMV-R and aGvHD were performed using Fisher’s exact test. Comparisons of cGvHD were performed using chi-square test. **Abbreviations:** No., number; %, percentage; CMV-R, CMV-reactivation; aGvHD, acute graft-versus-host disease; cGvHD: chronic GvHD.

### Univariate analysis of parameters affecting the outcome

OS, DFS, NRM and CIR were determined at a follow-up of 1, 2 and 5 years after allo-SCT. The OS was 75% / 64% / 54%, the DFS was 72% / 59% / 50%, the NRM was 16% / 21% / 24% and the CIR was 13% / 19% / 26% until 1, 2 and 5 years, respectively, in the whole cohort. The potential impact of the presence of CMV-CTLs at 3 months after allo-SCT on the outcome at 1, 2 and 5 years after allo-SCT was studied in univariate analysis along with other potential prognostic factors including patient age, gender, diagnosis, disease status, cyto-/molecular genetics, stem cell source, donor type, conditioning, GvHD prophylaxis, CMV-R, aGvHD or cGvHD (**Table 3**). Table 3 summarizes parameters significantly correlated with OS, DFS, NRM and CIR in the univariate regression analysis. Factors not significant are shown in S1 Table.

**Table 3:** Univariate analysis of the parameters influencing the outcome after allo-SCT.

Para-meter	Variables	OS			DFS			NRM			CIR		
		HR	95% CI	p value	HR	95% CI	p value	HR	95% CI	p value	HR	95% CI	p value
**Disease status**	**standard / advanced**												
	1 year	1.16	0.54–2.52	0.699	1.30	0.63–2.70	0.475	1.06	0.40–2.82	0.910	1.59	0.54–4.68	0.400
	2 years	1.31	0.69–2.50	0.410	1.59	0.87–2.91	0.135	0.94	0.40–2.18	0.880	2.64	1.05–6.62	**0.039**
	5 years	1.39	0.78–2.46	0.264	1.52	0.88–2.62	0.131	0.90	0.41–1.99	0.790	2.19	1.02–4.70	**0.044**
**Donor type**	**matched / mismatched**												
	1 year	2.19	0.92–5.21	0.077	1.93	0.82–4.52	0.131	1.86	0.62–5.55	0.260	1.75	0.48–6.38	0.400
	2 years	2.49	1.2–5.16	**0.014**	2.46	1.23–4.92	**0.011**	2.80	1.19–6.60	**0.019**	1.47	0.48–4.43	0.500
	5 years	1.96	0.97–3.95	0.061	2.00	1.02–3.91	**0.043**	2.41	1.03–5.63	**0.043**	1.02	0.34–3.07	0.980
**GvHD** **prophy-laxis**	**CsA-MTX / CsA-MMF**												
	1 year	2.05	0.70-.5.94	0.188	2.37	0.82–6.81	0.109	2.53	0.59–10.87	0.210	1.94	0.44–8.57	0.380
	2 years	2.50	0.97–6.41	0.057	2.02	0.90–4.55	0.090	2.34	0.71–7.73	0.170	1.44	0.50–4.18	0.500
	5 years	2.34	1.05–5.22	**0.038**	1.99	0.97–4.09	0.060	1.49	0.58–3.81	0.410	2.10	0.73–6.05	0.170
**aGvHD**	**grade 0-I / II-IV**												
	1 year	3.69	1.71–7.97	**0.001**	3.08	1.48–6.41	**0.003**	4.57	1.73–12.06	**0.002**	1.29	0.40–4.14	0.670
	2 years	4.92	2.57–9.43	**<0.001**	3.90	2.12–7.18	**<0.001**	6.54	2.79–15.34	**<0.001**	1.26	0.48–3.26	0.640
	5 years	3.93	2.19–7.05	**<0.001**	3.24	1.82–5.68	**<0.001**	5.83	2.66–12.77	**<0.001**	0.83	0.33–2.11	0.700
**cGvHD**	**no / yes**												
	1 year	0.21	0.06–0.71	**0.012**	0.18	0.05–0.59	**0.005**	0.24	0.06–1.07	0.061	0.14	0.02–1.11	0.063
	2 years	0.21	0.08–0.55	**<0.001**	0.21	0.09–0.51	**<0.001**	0.34	0.12–0.99	**0.047**	0.17	0.04–0.72	**0.016**
	5 years	0.26	0.12–0.53	**<0.001**	0.27	0.14–0.52	**<0.001**	0.51	0.22–1.19	0.120	0.22	0.08–0.61	**0.004**
**CMV-CTLs**	**no / yes**												
	1 year	1.00	0.34–2.89	0.994	0.51	0.22–1.19	0.120	2.89	0.38–22.03	0.300	0.18	0.06–0.53	**0.002**
	2 years	0.64	0.29–1.39	0.257	**0.48**	**0.24–0.98**	**0.045**	1.23	0.38–3.98	0.730	0.27	0.11–0.68	**0.005**
	5 years	0.61	0.30–1.22	0.161	0.61	0.30–1.21	0.159	1.43	0.44–4.64	0.560	0.40	0.16–1.00	0.050

Univariate regression analysis of OS and DFS were performed by Cox-regression/cox proportional hazard regression analysis. Analysis of NRM and CIR were performed by the Fine and Gray test. The second column shows for each tested parameter two alternative variables. For the calculation of the hazard ratio, the first variable was set as 1.00. **Abbreviations:** OS, overall survival; DFS, disease free survival; NRM, non-relapse mortality; CIR, cumulative incidence of relapse; HR, hazard ratio; CI, confidence interval; CSA, Cyclosporine A; MTX, methotrexate; MMF, mycophenolate mofetil; CMV-R, CMV reactivation; aGvHD, acute graft-versus-host disease; cGvHD: chronic GvHD.

**Fig 2:**
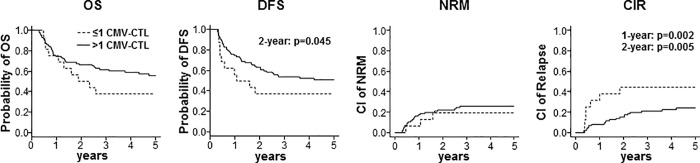
Presence of CMV-CTLs at 3 months after allo-SCT is associated with a reduced incidence of relapse. OS, DFS, NRM and CIR at a follow-up of 5 years are shown for all patients (n = 103) depending on the presence (black lines) or absence (dotted lines) of CMV-CTLs at 3 months after allo-SCT. Significant differences for the 2-year DFS (log-rank test) and the 1-, 2- and 5-year CIR (Gray’s test) between patients with CMV-CTLs and those without are indicated. Abbreviations: CI, cumulative incidence.

The OS was not affected by the presence of CMV-CTLs at 3 months after allo-SCT, but OS at 2 years was significantly longer in patients receiving matched donor grafts (p = 0.014) or at 5 years in patients receiving GvHD-prophylaxis with CsA/MTX (p = 0.038). The OS at 1, 2 and 5 years was also prolonged by the absence of aGvHD grade II-IV (p = 0.001, p<0.001 and p<0.001) and by the presence of cGvHD (p = 0.012, p<0.001 and p<0.001). The DFS at 2 years was significantly longer in patients with CMV-CTLs (p = 0.045, **Fig 2**). An improved DFS at 2 and 5 years was associated with matched donor grafts (p = 0.011 and p = 0.043). Additionally, an improved DFS at all time points analyzed was associated with the absence of aGvHD grade II-IV (p = 0.003, p<0.001 and p<0.001) and the presence of cGvHD (p = 0.005, p<0.001 and p<0.001). The NRM was not affected by the presence of CMV-CTLs by 3 months after allo-SCT. In contrast, an increased NRM at 2 and 5 years was associated with mismatched donor grafts (p = 0.019 and p = 0.043), at all time points with the presence of aGvHD grade II-IV (p = 0.002, p<0.001 and p<0.001) and at 2 years with the absence of cGvHD (p = 0.047). The CIR at 1 and 2 years was significantly lower in patients with CMV-CTLs (p = 0.002; p = 0.005; **Fig 2**). A reduced CIR at 2 and 5 years was also associated with a standard disease status (p = 0.039 and p = 0.044) and with cGvHD (p = 0.016; p = 0.004). There was a considerable difference in the time to relapse between patients with and without CMV-CTLs. At a 5-year follow up, only 65% of the relapses in CMV–CTL positive patients occurred until 2 years. In contrast, all relapses in CMV-CTL negative patients occurred until 2 years. Accordingly, onset of relapse was significantly later in CMV-CTL positive patients than in CMV-CTL negative patients (median 489 days, range 120–1532 vs. 152 days, range 129–668, p = 0.041, **Fig 2**).

### Multivariable regression analysis of parameters affecting the outcome

Multivariable regression analysis of the outcome was only performed with parameters statistically significant in the univariate analysis at 1, 2 and 5 years after allo-SCT (significant parameters in **Table 4**). The influence of the disease status on CIR was lost in the multivariable analysis (S2 Table). The OS at 1, 2 and 5 years was reduced by aGvHD grade II-IV (p = 0.001, p<0.001 and p<0.001). Chronic GvHD had a positive influence on OS at all time points analyzed (p = 0.009, p = 0.001 and p<0.001). The DFS at 2 and 5 years was significantly reduced in patients after mismatched transplantation (p = 0.049 and p = 0.038) and in patients with aGvHD grade II-IV (p = 0.001, p<0.001 and p<0.001) at all time points. In contrast, cGvHD led to an increase of the DFS at 1, 2 and 5 years (p = 0.003, p<0.001 p<0.001). The NRM at 1, 2 and 5 years was significantly increased by aGvHD grade II-IV (p = 0.002, p<0.001 and p<0.001) and at 2 years by the lack of cGvHD (p = 0.044). The CIR was only reduced at 1 and 2 years by the presence of CMV-CTLs (p = 0.006 and p = 0.039) and at 2 and 5 years by cGvHD (p = 0.04 and p = 0.02).

**Table 4:** Multivariable regression analysis of the parameters influencing the outcome after allo-SCT.

Para-meter	Variables	OS			DFS			NRM			CIR		
		HR	95% CI	p value	HR	95% CI	p value	HR	95% CI	p value	HR	95% CI	p value
**Donor type**	**matched/mismatched**												
	1 year		-			-			-			-	
	2 years	1.78	0.81–3.90	0.15	2.11	1.00–4.45	**0.049**	1.69	0.59–4.84	0.330		-	
	5 years				2.17	1.04–4.51	**0.038**	1.51	0.58–3.93	0.400		-	
**aGvHD**	**grade 0-I / II-IV**												
	1 year	3.95	1.82–8.55	**0.001**	3.34	1.59–6.99	**0.001**	4.69	1.79–12.27	**0.002**		**-**	
	2 years	4.66	2.32–9.36	**<0.001**	3.62	1.88–6.98	**<0.001**	5.71	2.16–15.12	**<0.001**		**-**	
	5 years	4.25	2.32–7.80	**<0.001**	3.14	1.71–5.76	**<0.001**	5.37	2.28–12.65	**<0.001**		**-**	
**cGvHD**	**no / yes**												
	1 year	0.20	0.06–0.66	**0.009**	0.17	0.05–0.55	**0.003**	0.24	0.05–1.09	0.065	0.17	0.02–1.31	0.088
	2 years	0.19	0.07–0.49	**0.001**	0.19	0.08–0.46	**<0.001**	0.34	0.12–0.97	0.044	0.21	0.05–0.93	**0.040**
	5 years	0.25	0.12–0.52	**<0.001**	0.22	0.11–0.44	**<0.001**		**-**		0.28	0.10–0.82	**0.020**
**CMV-CTLs**	**no / yes**												
	1 year		**-**			-			**-**		0.21	0.07–0.66	**0.006**
	2 years		**-**		0.82	0.39–1.73	0.600		-		0.35	0.14–0.95	**0.039**
	5 years		-			-			-			-	

Multivariable regression analysis of the outcome was performed only with those parameters statistically significant in the univariate analysis at 1, 2 or 5 years after allo-SCT. Multivariable regression analysis of OS and DFS were performed by Cox-regression/cox proportional hazard regression analysis. Analysis of NRM and CIR were performed by the Fine and Gray test. The second column shows for each tested parameter two alternative variables. Not significant data sets are indicated by”-”in the 95% CI column. For the calculation of the hazard ratio, the first variable was set as 1.00.

**Abbreviations**:”-“: not significant in univariate analysis. OS, overall survival; DFS, disease free survival; NRM, non-relapse mortality; CIR, cumulative incidence of relapse; HR, hazard ratio; CI, confidence interval; -, not applicable; aGvHD, acute graft-versus-host disease; cGvHD: chronic GvHD.

## Discussion

Our study is the first to show that the presence of CMV-CTLs at 3 months after allo-SCT in patients with hematological malignancies transplanted in the CMV R+/D+ setting is associated with a reduction of early relapses. The current study was prompted by our previous observations that the presence of CMV-CTLs patients in the CMV R+/D+ setting was associated with a fast T-cell reconstitution and elimination of the host hematopoiesis in patients with a broad spectrum of hematological diseases [20]. The latter data had suggested that CMV-CTLs might be a trigger for allo-immune responses reflected by the conversion to complete donor chimerism. The present study focused on the impact of CMV-CTLs in comparison to other demographic and clinical parameters on the outcome selectively in acute leukemia and MDS patients after allo-SCT. In accordance with previous reports [36], patients receiving a mismatched unrelated donor graft had a (borderline) reduced OS and a reduced DFS in the multivariable regression analysis. Severe aGvHD had no impact on the CIR but was associated with a reduced OS and DFS and an increased NRM.[7, 8] In accordance with previous reports, [7, 8] cGvHD was associated with an improved OS and DFS and a reduced CIR in the multivariable regression analysis. The advanced disease status [2, 3] and high-risk cyto- and molecular-genetics [4, 5] were significantly or by trend, respectively, associated with an increased CIR in the univariate analysis. However, this effect was lost in the multivariable analyses, maybe due to the still small sample size in our cohort (S2 Table). Taken together, our cohort compares well with other publications on the impact of the major disease or transplant related factors on the outcome. The most important finding of the current study was the association of the presence of CMV-CTLs with a reduced 1- and 2-year CIR both in the uni- and multivariable analysis. These data suggest that the previously observed suppression of host chimerism at 3 months after allo-SCT in patients with CMV-CTLs [20] translates at a longer follow-up in an anti-leukemic effect. Of note, the presence of CMV-CTLs at 3 months after transplantation and cGvHD were the only independent parameters significantly associated with a reduced CIR. However, while cGvHD was protective against late relapses, the protective effect of CMV-CTLs was restricted to early relapses after allo-SCT.

The mechanisms for the relationship between the presence of CMV-CTLs and prevention of early relapse remain unclear. CMV-R alone had no impact on the relapse incidence in our study. This may be due to the fact that in the previous studies on the protective effect of CMV-R against relapse the cohorts were heterogeneous regarding the CMV serostatus of patients and donors[13–18]. Here we studied the role of CMV-R selectively in the CMV R+/D+ setting alone, thus the impact of CMV-R is possibly less pronounced. Nevertheless, presence of CMV-CTLs at 3 months after allo-SCT was associated with prior CMV-R **(Table 2 and S1 Fig)** thus boosting the emergence of CMV-CTLs in the CMV +/+ setting^27^. Therefore, the presence of CMV-CTLs after CMV-R may be the key of protection against early relapse. Similar to our previous report [20], we found a strong association between presence of CMV-CTLs and a fast recovery of CD3+, CD8+ and CD4+ T-cells after allo-SCT also in the current cohort. Nevertheless, whether the presence of CMV-CTLs is solely a marker for a functional donor immune system becoming effective enough already at early time points after allo-SCT to protect from relapse or whether CMV-CTLs themselves play a causal role in promoting an effective anti-leukemic immune response remains speculative at present. Here, we analyzed the presence of CMV-CTLs in the context of clinical events. Functionality of the CMV-CTLS was not analyzed for this study, since tetramer staining alone does not give this information tetramer-staining of T-cells only indicates the presence of T-cells recognizing a CMV-peptide in context with a particular HLA-molecule, but is not sufficient to show the functionality of these T-cells. Additional tests such as intracellular cytokine staining or cytokine capture assay for IFN-gamma or IL-2 upon specific stimulation with CMV-peptides may quantify the functionality of the detected CMV-CTLs. Expansion of CMV-CTLs after CMV-reactivation/infection is a good surrogate marker for functionality of CMV-CTLs. [27] Furthermore, adoptive CMV-CTL transfer also increases overall CD8+ and CD4+ T-cell counts, [37, 38] indicating that CMV-CTLs might be a cause and not simply the consequence of an enhanced T-cell recovery. Moreover, the associations between anti-viral immune responses, allo-reactivity and lower leukemia relapse risk are most likely not limited to CMV. Own preliminary data reported previously [20] showed—at least by trend—an association between EBV-CTLs in HLA-A02+ patients and T-cell reconstitution even after restricting the analysis to patients without CMV-CTLs as potential confounding factor. Additionally, presence of T-cell responses directed against a broad spectrum of herpes viruses was associated with alloreavtive T-cell responses in pediatric patients after umbilical cord blood transplantation[39] and protective against leukemia relapse in high risk AML patients [40]. However, what links herpes viruses as immunological targets to antigens/peptides expressed on leukemia cells? Although cross-reactivity with antigens expressed on leukemia cells has been described [41], the targets of the virus-specific immune responses are most likely not expressed on leukemia cells. As discussed earlier [20], epithelial, myeloid and interstitial dendritic cells (DCs) [42, 43] are important reservoirs for latent CMV. Residual host-derived DCs are capable of presenting both host-alloantigens and CMV-antigens after allo-SCT. We hypothesize that the recognition of CMV on host DCs by CMV-CTLs may create a pro-inflammatory environment causing an enhanced presentation of host alloantigens [44]. Thereby, alloreactive donor T-cells might be boosted mediating the elimination of the residual leukemia cells [23]. This hypothesis is supported by the fact that the presence of CMV-CTL at 1, 2 and 3 month post-allo-SCT is associated with significantly higher counts of CD3+ T-cells in the patients (Fig 1). Alternatively, myeloid leukemia cells themselves may have been infected by CMV and CMV-antigens may directly be presented on the cell surface, thus direct elimination of leukemia cells by the CMV-CTLs may be possible as well. Future studies are required to proof either the proposed association between CMV-CTL and allo-antigen specific CTLs[23] by combined immune monitoring studies after allo-SCT. In addition, NK-cells, namely the subgroup of the NKG2C^+^CD57^+^ are reported to play important roles in reduction of CIR. We limited our studies to overall T cells and CMV-CTLs. A recent review by Litjens and colleagues [45] excellently summarizes several papers dealing with the impact CMV-R on CIR and the possible role of NK cells in both allo-SCT and solid organ transplantation. Interestingly, many studies describe an impact of early CMV-R on significantly reduced CIR in different cohorts. The reduction of relapse risk was associated with CMV-R, while pre-transplant CMV- seropositivity actually was associated with an increased relapse risk. The reduction of CIR in all previously published studies was correlated to patients with AML transplanted following MAC regimens and without in vivo depletion of T/NK-cells or immunosuppressive antibodies, like ATG or Thymoglobulin. These are major differences to our cohort. We included MDS and ALL patients to the AML patients (n = 74) for this paper to obtain larger patient numbers for the univariate and multivariable analysis. In the cohort (n = 103) described here CMV-reactivation had no influence in univariate analysis on CIR or any of the other parameters analyzed (S1 Table). To put our findings in perspective to data of other groups [45] we have analyzed patients with only AML (n = 74, 10 patients without CMV-CTL at 3 month) and found that in this cohort CMV-R was significantly correlated to OS at 2 and 5 years after SCT and with DFS at 5 years in the univariate regression analysis (S3 Table), but this significance was lost in the multivariable analysis (S4 Table). CMV-R did not correlate with CIR in our AML-cohort this may be due to the comparatively small numbers of patients but also other major differences of the cohort. In addition to the inclusion of other diseases as AML, diseases that have not shown the same correlation of CMV-R to CIR reduction, our patients were mainly transplanted following RIC (60%). Furthermore, more than 88% of the patients received in vivo T cell depletion with either ATG or thymoglobulin, which may also interfere with the correlation of CMV-R to the reduction of CIR. While these factors may explain the differences between the studied cohorts, we none the less feel that we may have elucidated one possible mechanism of CIR reduction following CMV-R. CMV-R is a prerequisite for the development and expansion of CMV-CTL^27^. We have shown here that the expansion of overall T cells is fast and yields higher numbers in patients with CMV-CTL (Fig 1). We argue that CMV-R may provide the optimal environment for not only CMV-CTL expansion, but an increased expansion of overall, thus possibly allo-reactive T cells.In conclusion, we have shown for the first time in a retrospective analysis that the presence of CMV-CTLs three months after allo-SCT is associated with a reduced incidence of early relapses in the CMV R+/D+ setting. Additional studies in larger and prospective cohorts are required to confirm the current observations and to unravel the mechanisms of the potential protective effects of CMV-CTLs against relapse.

## Supporting information

S1 Table Univariate analyses of the parameters possibly influencing outcome after allo-SCT (not significant factors. Univariate regression analysis of the outcome in the whole cohort was performed at 1, 2 or 5 years after allo-SCT. Univariate regression analysis of OS and DFS were performed by Cox-regression/cox proportional hazard regression analysis. Here, non-significant parameters are summarized. Analysis of CIR and NRM were performed by the Fine and Gray test. The first column shows the tested variables in the respective parameters and the hazard ratio (HR) are calculated using the first variable as a reference and set to 1. symbol: -, no events and results cannot be calculated.
(DOCX)

S2 Table Factors not significant after multivariable analysis. Multivariate regression analysis of the outcome was performed only with those parameters statistically significant in the univariate analysis at 1, 2 or 5 years after allo-SCT. Standard or advanced disease was significant in univariate analysis for CIR, but this was lost in the multivariate analyses. Multivariate regression analysis of OS and DFS were performed by Cox-regression/cox proportional hazard regression analysis. Analysis of NRM and CIR were performed by the Fine and Gray test. The second column shows for each tested parameter two alternative variables. For the calculation of the hazard ratio, the first variable was set as 1.00. Here, factors significant in univariate analysis, which lost significance in multivariable analysis are shown.”-”indicates parameters not significant in univariate analysis. **Abbreviations:** HR, hazard ratio; CI, confidence interval; -, not applicable; CSA, Cyclosporine A; MMF, mycophenolate mofetil; CMV-R, CMV reactivation; aGvHD, acute graft-versus-host disease; cGvHD: chronic GvHD.
(DOCX)

S3 Table Univariate analysis of the parameters influencing the outcome after allo-SCT in only AML patients. Univariate regression analysis of the outcome in the AML-only cohort was performed at 1, 2 or 5 years after allo-SCT. Univariate regression analysis of OS and DFS were performed by Cox-regression/cox proportional hazard regression analysis. Here, non-significant parameters are summarized. Analysis of CIR and NRM were performed by the Fine and Gray test. The first column shows the tested variables in the respective parameters and the hazard ratio (HR) are calculated using the first variable as a reference and set to 1. symbol: -, no events and results cannot be calculated. **Abbreviations:** HR, hazard ratio; CI, confidence interval; -, not applicable; CSA, Cyclosporine A; MMF, mycophenolate mofetil; CMV-R, CMV reactivation; aGvHD, acute graft-versus-host disease; cGvHD: chronic GvHD. In S3 Table CMV-R is associated with OS at 2 and 5 years and with DFS at 5 years in the univariate analysis, this correlation was lost in the multivariate analysis (S4 Table)
(DOCX)

S4 Table Multivariable analysis of the parameters influencing the outcome after allo-SCT in only AML patients. Multivariable regression analysis of the AML-only cohort for outcome was performed only with those parameters statistically significant in the univariate analysis at 1, 2 or 5 years after allo-SCT. Multivariate regression analysis of OS and DFS were performed by Cox-regression/cox proportional hazard regression analysis. Analysis of NRM and CIR were performed by the Fine and Gray test. The second column shows for each tested parameter two alternative variables. For the calculation of the hazard ratio, the first variable was set as 1.00. Here, factors significant in univariate analysis, which lost significance in multivariable analysis are shown.”-”indicates parameters not significant in univariate analysis. **Abbreviations:** HR, hazard ratio; CI, confidence interval; -, not applicable; CSA, Cyclosporine A; MMF, mycophenolate mofetil; CMV-R, CMV reactivation; aGvHD, acute graft-versus-host disease; cGvHD: chronic GvHD.
(DOCX)

S1 Fig CMV-R influences the presence of CMV CTLs until 3 months after allo-SCT. Depicted is the relationship between the presence or absence of CMV-R and the positivity for CMV CTLs at 1, 2 or 3 months after allo-SCT. The bars indicate % patients with >1 CMV-CTL/μl in patients without (open bars) or with (filled bars) CMV-R. Statistical analysis between groups at the respective months was performed by Fisher’s exact test.
(TIF)
