# Supplementary material for: Cytomegalovirus-specific CD8+ T-cells are associated with a reduced incidence of early relapse after allogeneic stem cell transplantation
Source: PLoS One. 2019 Mar 19;14(3):e0213739. doi: 10.1371/journal.pone.0213739 (PMC6424430; doi:10.1371/journal.pone.0213739)
Supplement: S1 Table — Univariate regression analysis of the outcome in the whole cohort was performed at 1, 2 or 5 years after allo-SCT. Univariate regression analysis of OS and DFS were performed by Cox-regression/cox proportional hazard regression analysis. Here, non-significant parameters are summarized. Analysis of CIR and NRM were performed by the Fine and Gray test. The first column shows the tested variables in the respective parameters and the hazard ratio (HR) are calculated using the first variable as a reference and set to 1. symbol: -, no events and results cannot be calculated. (DOCX) [file pone.0213739.s001.docx]

|  | **OS** |  |  | **DFS** |  |  | **NRM** |  |  | **CIR** |  |  |
| --- | --- | --- | --- | --- | --- | --- | --- | --- | --- | --- | --- | --- |
| **Parameter** | **HR** | **95% C.I** | **p value** | **HR** | **95% C.I** | **p value** | **HR** | **95% C.I** | **p value** | **HR** | **95% C.I** | **p value** |
| **≤60y / >60y** |  |  |  |  |  |  |  |  |  |  |  |  |
| 1 year | 1,32 | 0.59-2.96 | 0,502 | 1,29 | 0.60-2.76 | 0,521 | 1,48 | 0.54-4.06 | 0,440 | 1,03 | 0.32-3.28 | 0,970 |
| 2 years | 1,20 | 0.66-2.38 | 0,608 | 1,24 | 0.65-2.35 | 0,512 | 1,42 | 0.60-3.36 | 0,430 | 0,99 | 0.39-2.56 | 0,990 |
| 5 years | 1,31 | 0.72-2.40 | 0,380 | 1,22 | 0.68-2.18 | 0,497 | 1,17 | 0.50-2.73 | 0,720 | 1,17 | 0.53-2.56 | 0,700 |
| **male/female** |  |  |  |  |  |  |  |  |  |  |  |  |
| 1 year | 1,39 | 0.64-3.01 | 0,400 | 1,48 | 0.72-3.07 | 0,289 | 1,61 | 0.61-4.25 | 0,340 | 1,33 | 0.45-3.90 | 0,600 |
| 2 years | 1,11 | 0.58-2.14 | 0,751 | 1,10 | 0.60-2.05 | 0,752 | 1,36 | 0.59-3.13 | 0,470 | 0,84 | 0.34-2.08 | 0,700 |
| 5 years | 0,92 | 0.51-1.66 | 0,775 | 1,02 | 0.58-1.79 | 0,937 | 1,30 | 0.59-2.83 | 0,520 | 0,77 | 0.35-1.71 | 0,520 |
| **Acute leukemia* no / yes** |  |  |  |  |  |  |  |  |  |  |  |  |
| 1 year | 1,70 | 0.40-7.20 | 0,470 | 1,30 | 0.39-4.29 | 0,669 | 0,95 | 0.23-3.95 | 0,940 | 1,70 | 0.24-11.92 | 0,590 |
| 2 years | 2,58 | 0.62-10.75 | 0,192 | 1,43 | 0.51-4.00 | 0,498 | 1,35 | 0.32-5.74 | 0,680 | 1,28 | 0.32-5.10 | 0,730 |
| 5 years | 1,27 | 0.50-3.22 | 0,610 | 1,44 | 0.57-3.61 | 0,443 | 0,99 | 0.31-3.18 | 0,990 | 1,80 | 0.44-7.46 | 0,420 |
| **MDS no / yes** |  |  |  |  |  |  |  |  |  |  |  |  |
| 1 year | 0,59 | 0.14-2.49 | 0,470 | 0,77 | 0.23-2.55 | 0,669 | 1,05 | 0.25-4.37 | 0,940 | 0,59 | 0.08-4.14 | 0,590 |
| 2 years | 0,39 | 0.09-1.61 | 0,192 | 0,70 | 0.25-1.96 | 0,498 | 0,74 | 0.17-3.13 | 0,680 | 0,78 | 0.20-3.13 | 0,730 |
| 5 years | 0,79 | 0.31-1.99 | 0,610 | 0,70 | 0.28-1.75 | 0,443 | 1,01 | 0.31-3.24 | 0,990 | 0,55 | 0.13-2.29 | 0,420 |
| **Cyto-molecular genetics standard / high-risk** |  |  |  |  |  |  |  |  |  |  |  |  |
| 1 year | 1,04 | 0.47-2.28 | 0,931 | 0,96 | 0.45-2.03 | 0,914 | 0,94 | 0.35-2.58 | 0,910 | 0,99 | 0.33-2.99 | 0,980 |
| 2 years | 1,43 | 0.75-2.74 | 0,276 | 1,51 | 0.82-2.76 | 0,185 | 1,33 | 0.58-3.05 | 0,500 | 1,62 | 0.68-3.86 | 0,280 |
| 5 years | 1,67 | 0.94-2.96 | 0,080 | 1,62 | 0.94-2.79 | 0,085 | 1,27 | 0.58-2.77 | 0,550 | 1,79 | 0.85-3.77 | 0,130 |
| **BM/PBSC** |  |  |  |  |  |  |  |  |  |  |  |  |
| 1 year | - | - | - | - | - | - | - | - | - | - | - | - |
| 2 years | 1,66 | 0.23-12.10 | 0,618 | 1,96 | 0.27-14.26 | 0,506 | 0,90 | 0.15-5.62 | 0,910 | - | - | - |
| 5 years | 2,24 | 0.31-16.24 | 0,426 | 2,56 | 0.35-18.50 | 0,353 | 1,02 | 0.16-6.66 | 0,980 | - | - | - |
| **MAC/RIC** |  |  |  |  |  |  |  |  |  |  |  |  |
| 1 year | 1,26 | 0.56-2.83 | 0,572 | 1,23 | 0.57-2.64 | 0,599 | 2,00 | 0.65-6.13 | 0,230 | 0,73 | 0.25-2.14 | 0,560 |
| 2 years | 1,24 | 0.63-2.43 | 0,534 | 1,20 | 0.64-2.26 | 0,570 | 1,45 | 0.60-3.49 | 0,410 | 0,94 | 0.39-2.29 | 0,890 |
| 5 years | 1,47 | 0.79-2.71 | 0,221 | 1,41 | 0.79-2.51 | 0,248 | 1,20 | 0.54-2.67 | 0,650 | 1,52 | 0.66-3.50 | 0,330 |
| **noATG/ ATG** |  |  |  |  |  |  |  |  |  |  |  |  |
| 1 year | 1,47 | 0.35-6.20 | 0,603 | 1,73 | 0.41-7.29 | 0,453 | 0,82 | 0.19-3.56 | 0,790 | - | - | - |
| 2 years | 2,25 | 0.54-9.34 | 0,266 | 2,72 | 0.66-11.26 | 0,168 | 1,18 | 0.27-5.18 | 0,830 | - | - | - |
| 5 years | 1,45 | 0.52-4.03 | 0,482 | 1,71 | 0.62-4.76 | 0,301 | 0,62 | 0.23-1.69 | 0,350 | - | - | - |
| **No CMV-R / CMV-R** |  |  |  |  |  |  |  |  |  |  |  |  |
| 1 year | 2,43 | 0.97-6.05 | 0,057 | 1,87 | 0.88-4.22 | 0,133 | 2,07 | 0.69-6.23 | 0,200 | 1,44 | 0.44-4.65 | 0,550 |
| 2 years | 2,04 | 0.99-4.21 | 0,054 | 1,48 | 0.78-2.82 | 0,230 | 1,87 | 0.75-4.66 | 0,180 | 0,97 | 0.40-2.35 | 0,950 |
| 5 years | 1,65 | 0.89-3.05 | 0,108 | 1,56 | 0.87-2.79 | 0,132 | 1,82 | 0.78-4.25 | 0,170 | 1,11 | 0.51-2.39 | 0,800 |
