# Supplementary material for: Cytomegalovirus-specific CD8+ T-cells are associated with a reduced incidence of early relapse after allogeneic stem cell transplantation
Source: PLoS One. 2019 Mar 19;14(3):e0213739. doi: 10.1371/journal.pone.0213739 (PMC6424430; doi:10.1371/journal.pone.0213739)
Supplement: S3 Table — Univariate regression analysis of the outcome in the AML-only cohort was performed at 1, 2 or 5 years after allo-SCT. Univariate regression analysis of OS and DFS were performed by Cox-regression/cox proportional hazard regression analysis. Here, non-significant parameters are summarized. Analysis of CIR and NRM were performed by the Fine and Gray test. The first column shows the tested variables in the respective parameters and the hazard ratio (HR) are calculated using the first variable as a reference and set to 1. symbol: -, no events and results cannot be calculated. Abbreviations: HR, hazard ratio; CI, confidence interval; -, not applicable; CSA, Cyclosporine A; MMF, mycophenolate mofetil; CMV-R, CMV reactivation; aGvHD, acute graft-versus-host disease; cGvHD: chronic GvHD. In S3 Table CMV-R is associated with OS at 2 and 5 years and with DFS at 5 years in the univariate analysis, this correlation was lost in the multivariate analysis (S4 Table) (DOCX) [file pone.0213739.s003.docx]

| **Parameter** | **Variables** | **OS** | | | **DFS** | | | **NRM** | | | **CIR** | | |
| --- | --- | --- | --- | --- | --- | --- | --- | --- | --- | --- | --- | --- | --- |
|  |  | **HR** | **95% CI** | **p value** | **HR** | **95% CI** | **p value** | **HR** | **95% CI** | **p value** | **HR** | **95% CI** | **p value** |
| **Patient Age** | **≤60years/>60years** |  |  |  |  |  |  |  |  |  |  |  |  |
|  | 1 year | 2,47 | 0.89-6.79 | 0,082 | 2,13 | 0.80-5.67 | 0,132 | 2,04 | 0.60-6.91 | 0,250 | 1,88 | 0.39-9.09 | 0,430 |
|  | 2 years | 2,22 | 0.96-5.13 | 0,061 | 1,73 | 0.79-3.82 | 0,174 | 2,06 | 0.73-5.76 | 0,170 | 1,10 | 0.32-3.73 | 0,880 |
|  | 5 years | 1,87 | 0.90-3.90 | 0,093 | 1,57 | 0.79-3.11 | 0,195 | 1,62 | 0.61-4.30 | 0,340 | 1,22 | 0.48-3.12 | 0,680 |
| **Gender** | **male/female** |  |  |  |  |  |  |  |  |  |  |  |  |
|  | 1 year | 1,30 | 0.47-3.60 | 0,609 | 1,49 | 0.56-3.98 | 0,423 | 0,97 | 0.28-3.39 | 0,970 | 2,96 | 0.55-16.09 | 0,210 |
|  | 2 years | 1,25 | 0.54-2.90 | 0,601 | 1,19 | 0.54-2.62 | 0,665 | 1,09 | 0.39-3.10 | 0,870 | 1,26 | 0.39-4.05 | 0,700 |
|  | 5 years | 0,77 | 0.36-1.65 | 0,500 | 0,94 | 0.47-1.88 | 0,857 | 0,88 | 0.32-2.39 | 0,800 | 0,95 | 0.37-2.45 | 0,920 |
| **Disease status** | **standard/advanced** |  |  |  |  |  |  |  |  |  |  |  |  |
|  | 1 year | 1,38 | 0.50-3.79 | 0,536 | 1,60 | 0.60-4.30 | 0,351 | 1,19 | 0.35-4.02 | 0,780 | 2,40 | 0.45-12.91 | 0,310 |
|  | 2 years | 1,49 | 0.65-3.46 | 0,349 | 1,65 | 0.75-3.63 | 0,217 | 0,90 | 0.32-2.55 | 0,840 | 3,35 | 0.89-12.57 | 0,073 |
|  | 5 years | 1,38 | 0.66-2.85 | 0,390 | 1,52 | 0.78-2.99 | 0,223 | 0,92 | 0.35-2.45 | 0,870 | 2,11 | 0.83-5.34 | 0,120 |
| **Cyto- molecular genetics** | **standard/high-risk** |  |  |  |  |  |  |  |  |  |  |  |  |
|  | 1 year | 0,55 | 0.16-1.96 | 0,357 | 0,50 | 0.14-1.75 | 0,275 | 0,54 | 0.12-2.56 | 0,440 | 0,44 | 0.05-3.79 | 0,450 |
|  | 2 years | 1,26 | 0.53-3.01 | 0,598 | 1,24 | 0.55-2.81 | 0,606 | 1,21 | 0.42-3.50 | 0,730 | 1,28 | 0.38-4.26 | 0,690 |
|  | 5 years | 1,37 | 0.65-2.90 | 0,414 | 1,44 | 0.72-2.87 | 0,306 | 1,00 | 0.36-2.82 | 1,000 | 1,85 | 0.75-4.56 | 0,180 |
| **Stem cell source** | **BM/PBSC** |  |  |  |  |  |  |  |  |  |  |  |  |
|  | 1 year | **-** | **-** | **-** | **-** | **-** | **-** | **-** | **-** | **-** | **-** | **-** | **-** |
|  | 2 years | 0,94 | 0.13-6.99 | 0,951 | 1,08 | 0.15-7.98 | 0,940 | 0,56 | 0.09-3.39 | 0,530 | **-** | **-** | **-** |
|  | 5 years | 1,30 | 0.18-9.60 | 0,795 | 1,53 | 0.21-11.2 | 0,675 | 0,63 | 0.10-4.05 | 0,630 | **-** | **-** | **-** |
| **Donor type** | **matched/mismatched donors** |  |  |  |  |  |  |  |  |  |  |  |  |
|  | 1 year | 1,95 | 0.55-6.92 | 0,301 | 1,86 | 0.53-6.55 | 0,332 | 1,83 | 0.42-7.91 | 0,420 | 1,55 | 0.17-13.86 | 0,690 |
|  | 2 years | 2,52 | 0.93-6.83 | 0,071 | 2,89 | 1.15-7.26 | **0,024** | 3,14 | 1.08-9.16 | **0,036** | 1,72 | 0.37-7.91 | 0,490 |
|  | 5 years | 1,99 | 0.75-5.25 | 0,165 | 2,21 | 0.91-5.36 | 0,081 | 2,71 | 0.93-7.92 | 0,068 | 0,98 | 0.21-4.52 | 0,980 |
| **Conditioning** | **MAC/RIC** |  |  |  |  |  |  |  |  |  |  |  |  |
|  | 1 year | 3,24 | 0.73-14.36 | 0,122 | 3,49 | 0.80-15.4 | 0,098 | 4,28 | 0.54-33.94 | 0,170 | 2,32 | 0.29-19.35 | 0,440 |
|  | 2 years | 3,33 | 0.99-11.27 | 0,053 | 2,16 | 0.81-5.77 | 0,124 | 2,95 | 0.67-12.91 | 0,150 | 1,27 | 0.35-4.60 | 0,710 |
|  | 5 years | 3,54 | 1.23-10.18 | **0,019** | 2,67 | 1.10-6.45 | **0,029** | 2,17 | 0.64-7.33 | 0,210 | 2,44 | 0.70-8.47 | 0,160 |
| **T cell depleting antibodies**** | **no/yes** |  |  |  |  |  |  |  |  |  |  |  |  |
|  | 1 year | 1,97 | 0.26-15.02 | 0,511 | 2,18 | 0.30-16.51 | 0,450 | 1,24 | 0.16-9.84 | 0,840 | **-** | **-** | **-** |
|  | 2 years | 3,13 | 0.42-23.31 | 0,265 | 3,74 | 0.51-27.65 | 0,196 | 1,82 | 0.23-14.53 | 0,570 | **-** | **-** | **-** |
|  | 5 years | 2,19 | 0.52-9.23 | 0,285 | 2,73 | 0.65-11.41 | 0,169 | 1,00 | 0.24-4.17 | 1,000 | **-** | **-** | **-** |
| **GvHD prophylaxis** | **CsA,MTX/CsA,MMF** |  |  |  |  |  |  |  |  |  |  |  |  |
|  | 1 year | 2,62 | 0.59-11.62 | 0,205 | 2,83 | 0.64-12.46 | 0,169 | 3,48 | 0.44-27.64 | 0,240 | 1,90 | 0.23-15.78 | 0,550 |
|  | 2 years | 2,67 | 0.79-9.04 | 0,114 | 1,73 | 0.65-4.61 | 0,275 | 2,39 | 0.55-10.45 | 0,250 | 1,04 | 0.29-3.73 | 0,960 |
|  | 5 years | 2,81 | 0.98-8.07 | 0,056 | 2,10 | 0.87-5.07 | 0,100 | 1,75 | 0.52-5.91 | 0,370 | 1,97 | 0.57-6.84 | 0,280 |
| **CMV-R** | **no/yes** |  |  |  |  |  |  |  |  |  |  |  |  |
|  | 1 year | 2,75 | 0.78-9.74 | 0,118 | 2,91 | 0.83-10.21 | 0,096 | 2,62 | 0.58-11.88 | 0,210 | 3,07 | 0.35-26.73 | 0,310 |
|  | 2 years | 3,24 | 1.10-9.57 | **0,033** | 2,31 | 0.92-5.78 | 0,075 | 2,45 | 0.70-8.52 | 0,160 | 1,72 | 0.47-6.31 | 0,410 |
|  | 5 years | 2,40 | 1.03-5.63 | **0,044** | 2,16 | 1.01-4.64 | **0,048** | 2,03 | 0.68-6.09 | 0,200 | 1,73 | 0.63-4.74 | 0,280 |
| **aGvHD** | **grade 0-I/ II-IV** |  |  |  |  |  |  |  |  |  |  |  |  |
|  | 1 year | 3,92 | 1.42-10.82 | **0,008** | 3,43 | 1.28-9.16 | **0,014** | 4,99 | 1.46-17.05 | **0,010** | 1,37 | 0.26-7.26 | 0,720 |
|  | 2 years | 5,48 | 2.33-12.86 | **<0.001** | 4,20 | 1.90-9.24 | **<0.001** | 6,19 | 2.13-17.97 | **0,001** | 1,62 | 0.48-5.46 | 0,430 |
|  | 5 years | 4,11 | 1.96-8.60 | **<0.001** | 3,04 | 1.53-6.06 | **0,002** | 5,90 | 2.2-15.84 | **<0.001** | 0,81 | 0.26-2.53 | 0,720 |
| **cGvHD** | **no/yes** |  |  |  |  |  |  |  |  |  |  |  |  |
|  | 1 year | 0,10 | 0.01-0.73 | **0,024** | 0,09 | 0.01-0.66 | **0,018** | 0,16 | 0.02-1.22 | 0,077 | **-** | **-** | **-** |
|  | 2 years | 0,12 | 0.03-0.53 | **0,005** | 0,16 | 0.05-0.54 | **0,003** | 0,23 | 0.05-1.00 | **0,050** | 0,14 | 0.02-1.05 | 0,055 |
|  | 5 years | 0,24 | 0.09-0.64 | **0,004** | 0,28 | 0.12-0.64 | **0,003** | 0,45 | 0.15-1.33 | 0,150 | 0,26 | 0.08-0.87 | **0,029** |
| **CMV-CTLs** | **negative/positive** |  |  |  |  |  |  |  |  |  |  |  |  |
|  | 1 year | 1,02 | 0.23-4.50 | 0,984 | 0,64 | 0.18-2.23 | 0,481 | 1,42 | 0.18-11.10 | 0,740 | 0,30 | 0.06-1.55 | 0,150 |
|  | 2 years | 0,51 | 0.19-1.38 | 0,184 | 0,55 | 0.21-1.46 | 0,228 | 0,57 | 0.17-1.89 | 0,360 | 0,65 | 0.14-3.07 | 0,590 |
|  | 5 years | 0,68 | 0.26-1.79 | 0,436 | 0,78 | 0.30-2.01 | 0,606 | 0,66 | 0.20-2.21 | 0,500 | 1,19 | 0.25-5.59 | 0,830 |
