# Supplementary material for: Cytomegalovirus-specific CD8+ T-cells are associated with a reduced incidence of early relapse after allogeneic stem cell transplantation
Source: PLoS One. 2019 Mar 19;14(3):e0213739. doi: 10.1371/journal.pone.0213739 (PMC6424430; doi:10.1371/journal.pone.0213739)
Supplement: S4 Table — Multivariable regression analysis of the AML-only cohort for outcome was performed only with those parameters statistically significant in the univariate analysis at 1, 2 or 5 years after allo-SCT. Multivariate regression analysis of OS and DFS were performed by Cox-regression/cox proportional hazard regression analysis. Analysis of NRM and CIR were performed by the Fine and Gray test. The second column shows for each tested parameter two alternative variables. For the calculation of the hazard ratio, the first variable was set as 1.00. Here, factors significant in univariate analysis, which lost significance in multivariable analysis are shown.”-”indicates parameters not significant in univariate analysis. Abbreviations: HR, hazard ratio; CI, confidence interval; -, not applicable; CSA, Cyclosporine A; MMF, mycophenolate mofetil; CMV-R, CMV reactivation; aGvHD, acute graft-versus-host disease; cGvHD: chronic GvHD. (DOCX) [file pone.0213739.s004.docx]

| **Parameter** | **Variables** | **OS** | | | **DFS** | | | **NRM** | | | **CIR** | | |
| --- | --- | --- | --- | --- | --- | --- | --- | --- | --- | --- | --- | --- | --- |
|  |  | **HR** | **95% CI** | **p value** | **HR** | **95% CI** | **p value** | **HR** | **95% CI** | **p value** | **HR** | **95% CI** | **p value** |
| **Donor type** | matched/mismatched donors |  |  |  |  |  |  |  |  |  |  |  |  |
|  | 1 year |  |  |  |  |  |  |  |  |  |  |  |  |
|  | 2 years |  |  |  | 2,54 | 0.94-6.88 | 0,066 | 1,87 | 0.46-7.70 | 0,380 |  |  |  |
|  | 5 years |  |  |  |  |  |  |  |  |  |  |  |  |
| **Conditioning** | MAC/RIC |  |  |  |  |  |  |  |  |  |  |  |  |
|  | 1 year |  |  |  |  |  |  |  |  |  |  |  |  |
|  | 2 years |  |  |  |  |  |  |  |  |  |  |  |  |
|  | 5 years | 2,92 | 0.92-9.25 | 0,069 | 2,41 | 0.92-6.33 | 0,075 |  |  |  |  |  |  |
| **CMV-R** | no/yes |  |  |  |  |  |  |  |  |  |  |  |  |
|  | 1 year |  |  |  |  |  |  |  |  |  |  |  |  |
|  | 2 years | 2,92 | 0.98-8.67 | 0,054 |  |  |  |  |  |  |  |  |  |
|  | 5 years | 1,46 | 0.58-3.71 | 0,425 | 1,38 | 0.60-3.19 | 0,451 |  |  |  |  |  |  |
| **aGvHD** | grade 0-I/ II-IV |  |  |  |  |  |  |  |  |  |  |  |  |
|  | 1 year | 4,30 | 1.55-11.93 | **0,005** | 3,76 | 1.40-10.09 | **0,009** |  |  |  |  |  |  |
|  | 2 years | 6,92 | 2.90-16.53 | **<0.001** | 4,16 | 1.76-9.83 | **0,001** | 5,50 | 1.51-20.11 | **0,010** |  |  |  |
|  | 5 years | 5,99 | 2.69-13.34 | **<0.001** | 4,44 | 2.11-9.37 | **<0.001** |  |  |  |  |  |  |
| **cGvHD** | no/yes |  |  |  |  |  |  |  |  |  |  |  |  |
|  | 1 year | 0,09 | 0.01-0.68 | **0,020** | 0,08 | 0.01-0.62 | **0,015** |  |  |  |  |  |  |
|  | 2 years | 0,11 | 0.03-0.48 | **0,003** | 0,13 | 0.04-0.44 | **0,001** | 0,22 | 0.05-0.97 | **0,045** |  |  |  |
|  | 5 years | 0,20 | 0.07-0.55 | **0,002** | 0,23 | 0.10-0.56 | **0,001** |  |  |  | 0,26 | 0.08-0.87 | **0,029** |
