# Supplementary figures and images for: Cytomegalovirus-specific CD8+ T-cells are associated with a reduced incidence of early relapse after allogeneic stem cell transplantation
Source: PLoS One. 2019 Mar 19;14(3):e0213739. doi: 10.1371/journal.pone.0213739 (PMC6424430; doi:10.1371/journal.pone.0213739)

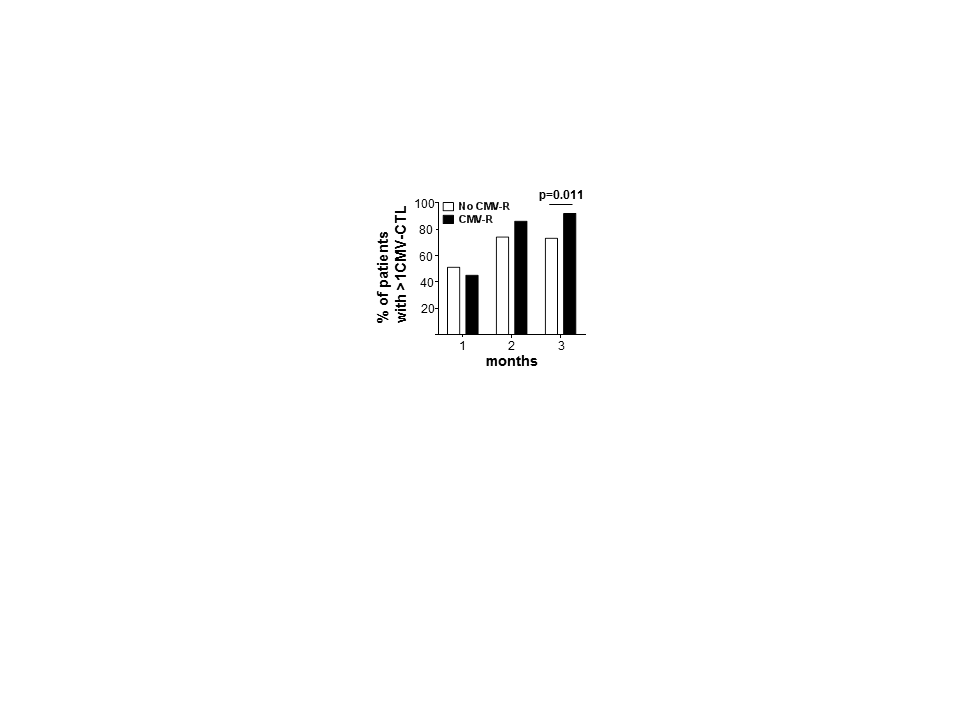

Supplement: S1 Fig — Depicted is the relationship between the presence or absence of CMV-R and the positivity for CMV CTLs at 1, 2 or 3 months after allo-SCT. The bars indicate % patients with >1 CMV-CTL/μl in patients without (open bars) or with (filled bars) CMV-R. Statistical analysis between groups at the respective months was performed by Fisher’s exact test. (TIF) [file pone.0213739.s005.TIF]
